# The effectiveness and predictors influencing the outcome of onabotulinumtoxinA treatment in chronic migraine: understanding from diverse patient profiles in a single session

**DOI:** 10.3389/fneur.2024.1417303

**Published:** 2024-06-19

**Authors:** Elif Ilgaz Aydinlar, Tuba Erdogan Soyukibar, Pinar Yalinay Dikmen

**Affiliations:** Department of Neurology, Acıbadem University School of Medicine, Istanbul, Türkiye

**Keywords:** chronic migraine, onabotulinumtoxinA, outcome, anxiety, depression, bruxism, efficacy, predictors

## Abstract

**Objective:**

This real-world study aimed to investigate how onabotulinumtoxinA affects the outcome of migraine, along with accompanying anxiety, depression, and bruxism among a group of patients with chronic migraine (CM) and define predictors of good response.

**Methods:**

Patients diagnosed with CM who received onabotulinumtoxinA were included in this single-center, real-world retrospective cohort study. Monthly headache days (MHDs), monthly migraine days (MMDs), headache intensity (numeric rating scale-NRS) and headache characteristics were evaluated at baseline and 12 weeks post-treatment. Patient-reported outcome measures (PROMs) included Migraine Disability Assessment Scale (MIDAS), Headache Impact Test-6 (HIT-6) scores, 12-item Allodynia Symptom Checklist (ASC-12), Beck Anxiety Inventory (BAI) and Beck Depression Inventory (BDI). Response to onabotulinumtoxinA (% reduction in MHDs) and treatment-related adverse events (TRAEs) were also evaluated. OnabotulinumA was applied to the masseter muscles in patients complaining of bruxism.

**Results:**

A total of 72 patients (mean ± SD age: 36.3 ± 8.5 years; 91.7% were female) diagnosed with CM were included. OnabotulinumtoxinA revealed significant decrease in median (IQR) MHDs [from 20(15–25) at baseline to 6(4–10), *p* < 0.001], MMDs [from 9(6–12) to 3(1–6), *p* < 0.001] and NRS [from 9(8–10) to 7(6–8), *p* < 0.001], and the MIDAS [from 54(30–81) to 16(7–24), *p* < 0.001], HIT-6 [from 67(65–69) to 58(54–64), *p* < 0.001], ASC-12 [from 6(1.5–9) to 2(0–9), *p* = 0.002], BAI [from 12(6.5–19) to 9(3–17), *p* < 0.001] and BDI [from 11(6.5–17) to 3(2–7) *p* < 0.001] scores at 12 weeks post-treatment. Patients complaining of bruxism received onabotulinumtoxinA injections in the first *n* = 27 (37.5%) and 12. week post-treatment *n* = 19 (70.4%) periods. Overall, 70.8% of patients responded (≥50% reduction in MHDs), while 29.2% did not (<50% reduction). Both groups showed similar characteristics in demographics, migraine history, baseline PROMs scores, comorbidities, and prior treatments.

**Conclusion:**

OnabotulinumtoxinA is an effective treatment option that rapidly improves migraine outcomes, disability, and impact while also alleviating comorbid depression and/or anxiety. This study’s noteworthy finding is that onabotulinumtoxinA is effective in a majority of CM patients, irrespective of their prior treatment history, migraine characteristics, or concurrent comorbidities. Furthermore, we identified no specific predictors for a favorable response to onabotulinumtoxinA. Applying onabotulinumtoxinA to the masseter muscles can relieve discomfort associated with concurrent bruxism; however, it does not impact migraine outcomes.

## Introduction

Migraine, affecting 14% of the global population, is a severe condition leading to marked disability, compromised functionality, diminished quality of life for sufferers, and imposing a considerable socioeconomic load (1–3). Based on the monthly headache days (MHDs), migraine is classified as episodic (EM) and chronic migraine (CM) (4, 5). Patients with EM face the possibility of advancing to CM, while those with CM are at increased risk of migraine disability, impaired quality of life, comorbid medical and psychiatric conditions, and medication-overuse headache (MOH) (5–9). Migraine coexists with psychiatric comorbidities such as anxiety and depression, posing a risk of migraine chronification and poor treatment response (10–12). Sleep bruxism may cause morning headaches with moderate, non-pulsating, and pressure-like symptoms (13) and is found in CM as common as in EM patients but causes more disability (14). Hence, effective migraine management requires prophylactic treatment (15, 16) and addressing comorbidities due to their impact on treatment efficacy and clinical course (10, 17, 18). Despite the availability of several drugs for migraine prevention, studies indicate low adherence to oral migraine prophylactics, with adverse events being the most frequently cited reason for discontinuation, followed by lack of efficacy (19–21). This situation emphasizes the necessity for early, targeted preventive treatments that fulfill both patient and physician expectations with greater specificity and effectiveness (22–24).

Real-world evidence regarding the effectiveness and safety of onabotulinumtoxinA in treating CM is crucial, especially considering the diverse patient population with comorbidities and the complex clinical management strategies not fully represented in randomized controlled trials (RCTs) (25–27). Furthermore, due to the connection between the decrease in headache intensity and disability scales, conducting a thorough headache assessment with patient-reported outcome measures (PROMs), beyond tracking headache days, is advised for a more accurate evaluation of treatment response in chronic migraine. However, only some real-world studies have specifically examined the impact of onabotulinumtoxinA on the qualitative aspects of headache pain in treated individuals (28–30).

Despite being the subject of numerous studies over the past two decades, there still needs to be a consensus on the predictors in chronic migraine patients who respond well to onabotulinumtoxinA. Predictors indicating a favorable response have been suggested to include clinical headache characteristics such as unilateral or ocular location (31–34), level of disability, headache intensity (31), frequency of migraine days per month, and medication overuse (35). Additionally, molecular biomarkers such as elevated serum levels of calcitonin gene-related peptide (CGRP), vasoactive intestinal peptide, and pentraxin-3 have been found to aid in predicting a positive response to onabotulinumtoxinA (36, 37).

This study aims to see the effect of single-session onabotulinumtoxinA on migraine outcomes and accompanying comorbid diseases such as anxiety/depression and to predict which patient profile benefits from onabotulinumtoxinA. Furthermore, we aimed to assess whether implementing an additional injection protocol to alleviate concurrent bruxism in these patients had any impact on migraine recovery.

## Methods

### Study population

**Figure 1:**
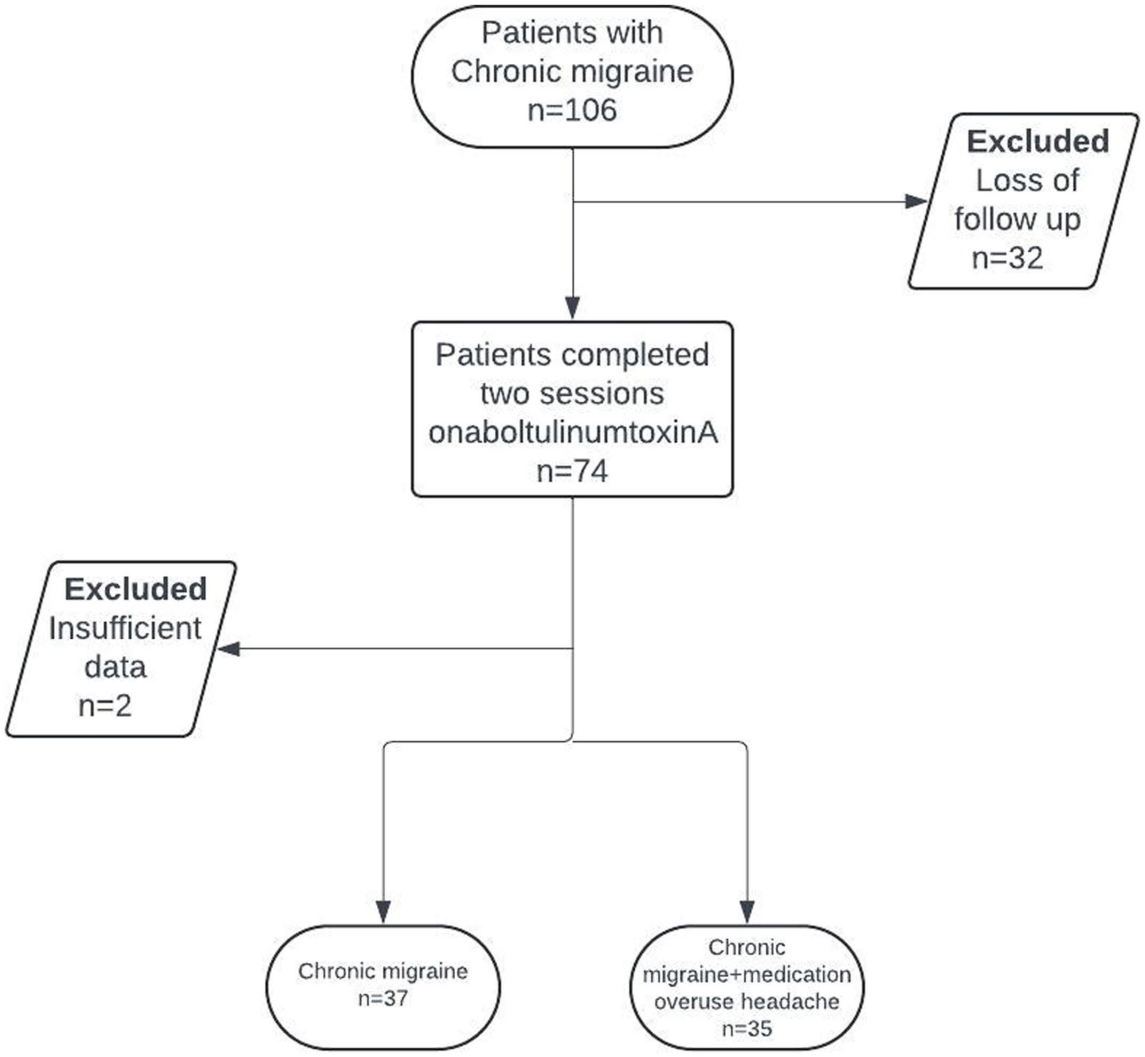
Flow chart of patient selection.

In this retrospective cohort study, patients between 18 and 65 diagnosed CM were included, according to the third edition of the International Classification of Headache Disorders (ICHD-3), who received a single-session onabotulinumtoxinA for migraine prophylaxis and had follow-up at 12 weeks (38). The patient selection for this study is depicted in Figure 1. Throughout the study duration and up to 1 month before enrollment, participants did not receive any other prophylactic migraine treatments, additional antidepressants, nerve blocks, or trigger point injections for migraine or any comorbid conditions. Exclusion criteria included pregnant or breastfeeding women, as well as individuals who had recently initiated a new psychiatric medication or undergone dose adjustments for ongoing psychiatric medication within the 3 months preceding the study enrollment.

This study was approved by our university’s Medical Research Ethics Committee (Approval number: 2023-21/726).

### Study parameters

Data on patient demographics (age, gender), educational status and employment status, family history for migraine, comorbid diseases, presence of medication overuse headache (MOH) and bruxism, duration of disease, migraine triggers, commonly used analgesics (acute migraine treatment) and previous migraine treatments were recorded at baseline. Migraine outcome, headache characteristics, accompanying symptoms including interictal (between attacks) and ictal (during an attack) photophobia (scored 1: none to 5: extreme disturbance), ictal phonophobia, and ictal osmophobia (yes and no), and PROMs were evaluated at baseline and 12—weeks post-treatment.

Migraine outcome was assessed based on MHDs, MMDs, headache intensity via a numeric rating scale (NRS) (graded 0: no pain to 10: the worst pain imaginable), and the days of analgesics (migraine-specific and non-specific) used in a month. PROMs included Migraine Disability Assessment Scale (MIDAS), Headache Impact Test-6 (HIT-6) scores, 12-item Allodynia Symptom Checklist (ASC-12), Beck Anxiety Inventory (BAI), and Beck Depression Inventory (BDI).

The treatment response to onabotulinumtoxinA was assessed based on the response rate (percentage reduction in monthly headache days), while safety outcomes, including treatment-related adverse events (TRAEs), were evaluated 12 weeks after treatment. Headache relief within 2 h was evaluated using a 5-point Likert scale to assess the response to acute treatment (ranging from 1, indicating never, to 5, indicating always).

### MIDAS

The MIDAS is a self-administered 5-item questionnaire used to quantitatively evaluate headache-related disability regarding the number of days in the past 3 months and activity limitations due to migraine. The final total score corresponds to the sum of missed days for the three activities. It is categorized depending on the severity of attacks as little or no disability (scores 0–5), mild disability (scores 6–10), moderate disability (scores 11 to 20) or severe disability (scores ≥ 21) (39, 40).

### HIT-6

HIT-6 is a 6-item questionnaire with domains on pain, social functioning, role functioning, vitality, cognitive functioning, and psychological distress. Each item is answered on a 5-point Likert scale (6 = never, 8 = rarely, 10 = sometimes, 11 = very often, 13 = always). The total score ranges between 36 and 78, with higher scores reflecting more significant impact, as categorized into four groups including little or no impact (scores ≤ 49), some impact (scores 50–55), substantial impact (scores 56–59) and, severe impact (scores ≥ 60) (41, 42).

### ASC-12

ASC-12 was used to assess allodynia on 12 items during headache; each scored as 0 (not apply to me, never, rarely), 1 (less than half of the time), or 2 (half of the time or more). The total score indicated the allodynia range, as categorized into none (scores 0–2), mild (scores 3–5), moderate (scores 6–8), and severe (scores ≥ 9) (43, 44).

### BDI

BDI is a 21-item self-reporting questionnaire for the assessment of the level and changes in the severity of depression over the past 2 weeks based on physical, emotional, cognitive, and motivational symptoms. Each item is scored on a 4-point scale from 0 (no symptom) to 3 (severe symptoms), and the total score achieved by adding the highest ratings for all 21 items ranges from 0 to 63, with higher scores indicating greater symptom severity. Based on the total score, individuals are categorized as severe (scores 30–63), moderate (scores 19–29), mild depression (scores 10–18), and none/minimal depression (scores 0–9) (45, 46).

### BAI

This 21-item scale is a self-report measure of anxiety. Each item is scored from 0 (not at all) to 3 (severely—it bothered me a lot), and the total score is calculated by finding the sum of the 21 items and classified as low (scores 0–21), moderate (scores 22–35) and severe anxiety (scores ≥ 36) (47, 48).

### OnabotulinumtoxinA injection protocol

Administration of onabotulinumtoxinA was performed as 31 fixed-site, fixed-dose intramuscular injections applied at seven specified head and neck muscle points at baseline and after 12 weeks (2 onabotulinumtoxinA sessions) according to the injection scheme proposed in the PREEMPT studies (49, 50). Additional injection sites involved occipitalis, temporalis, or trapezius muscles using a follow-the-pain strategy. During the 12-week interval, patients were asked to keep a headache diary.

Patients experiencing symptoms of bruxism, including jaw discomfort, nighttime clenching noted by partners, clenching during daytime, and morning headaches without migraine characteristics, were administered onabotulinumtoxinA doses ranging from 10 to 30 IU per masseter muscle. A previous diagnosis by a dentist, physical indications, including hypertrophy of the masseter muscles, linea alba on the cheek mucosa, or signs of pressure on the tongue, were considered supportive markers of bruxism. At the second visit, any reduction in bruxism symptoms was evaluated, and patients who reported improvement received additional injections to the masseter muscles, while those reporting no change did not undergo further treatment.

### Treatment response categories

Patients with ≥50% reduction of MHDs from baseline were considered responders, while non-responders were those with <50% reduction of MHDs from baseline. Patients with ≥75% reduction of MHDs from baseline were considered super-responders.

### Statistical analysis

Statistical analysis was conducted using MedCalc® Statistical Software version 19.7.2 (MedCalc Software Ltd., Ostend, Belgium; https://www.medcalc.org; 2021). The normality of continuous variables was assessed via Shapiro–Wilk’s test. Descriptive statistics included mean, standard deviation, median, and interquartile range for continuous variables and frequencies (*n*) and percentages (%) for categorical variables.

For non-normally distributed independent continuous data involving more than two groups, the Kruskal-Wallis Test was employed. For non-normally distributed dependent continuous data comparing two groups, the Wilcoxon Signed Rank Test was used. The Mann–Whitney U Test was applied when comparing two groups with non-normally distributed independent continuous data. A significance level of *p* < 0.005 was set.

## Results

### Baseline characteristics and migraine history

Seventy-four patients out of 106 completed both sessions of onabotulinumtoxinA treatment, while 2 patients were excluded due to insufficient documentation. The mean ± SD age of the 72 patients included was 36.3 ± 8.5 years, with 91.7% of them being female. Most of the patients were university graduates (86.1%), employed (75.0%), and had a migraine family history (65.3%). Thirty-five patients (48.6%) had MOH. Comorbidities included bruxism in 37.5% of patients (*n* = 27), sleep disorder in 37.5% (*n* = 27), chronic pain in 34.7% (*n* = 25), and psychiatric disease in 29.2% (*n* = 21; anxiety in 19.4% and depression in 16.6%; Table 1).

**Table 1:** Baseline characteristics and migraine disease and treatment history (*n* = 72).

Age (year), mean ± SD; median (IQR)	36.3 ± 8.5; 35(31–41)
**Gender, *n*(%)**	
*Female*	66(91.7)
*Male*	6(8.3)
**Educational status, *n*(%)**	
*High school*	10(13.9)
*University*	62(86.1)
**Employment, *n*(%)**	
*Unemployed*	18(25.0)
*Employed*	54(75.0)
Family history for migraine (yes), *n*(%)	47(65.3)
Medication Overuse Headache, *n*(%)	35(48.6)
Number of comorbidities, median (IQR)	2(1–3)
**Type of comorbidity, *n*(%)**	
*Bruxism*	27(37.5)
*Sleep disorder*	27(37.5)
*Chronic pain*	25(34.7)
*Psychiatric disease*	21(29.2)
*Anxiety*	14(19.4)
*Depression*	12(16.6)
Duration of migraine (years), median (IQR)	10.0(6–20)
**Migraine triggers (five most common), *n*(%)**	
*Stress*	63(87.5)
*Lack of sleep or excessive sleeping*	57(79.2)
*Bright light*	49(68.1)
*Change of barometric pressure*	47(65.3)
*Menstruation*	43(59.7)
Aura (visual), *n*(%)	8(11.1)
**Acute non-pharmacological headache relievers, *n*(%)**	
*Quiet and dark places*	55(76.4)
*Sleeping*	47(65.3)
**Analgesics, *n*(%)**	
*NSAIDs*	47(65.3)
*Triptan*	20(27.8)
*Paracetamol*	20(27.8)
*Combined analgesics*	10(13.9)
*Ergot*	7(9.7)
Response to analgesics, median (IQR)	3(2–4)
Previous prophylactic treatments, *n*(%)^*^	40(55.6)

IQR, Interquartile range; NSAIDs, Non-steroidal anti-inflammatory drugs. ^*^OnabotulinumtoxinA, antiepileptics, beta blockers, antidepressants.

Stress (87.5%), lack of sleep/excessive sleeping (79.2%), and bright lights (68.1%) were the most commonly reported migraine triggers, while quiet and dark places (76.4%) and sleeping (65.3%) were the most common acute non-pharmacological -headache relievers (Table 1).

Non-steroidal anti-inflammatory drugs (NSAIDs, 65.3%) were the most commonly used analgesics, while the response to acute treatment was noted as median (IQR) 3(2–4). The percentage of patients with prior migraine prophylaxis experience was 55.6% (Table 1).

### Migraine outcome

**Figure 2:**
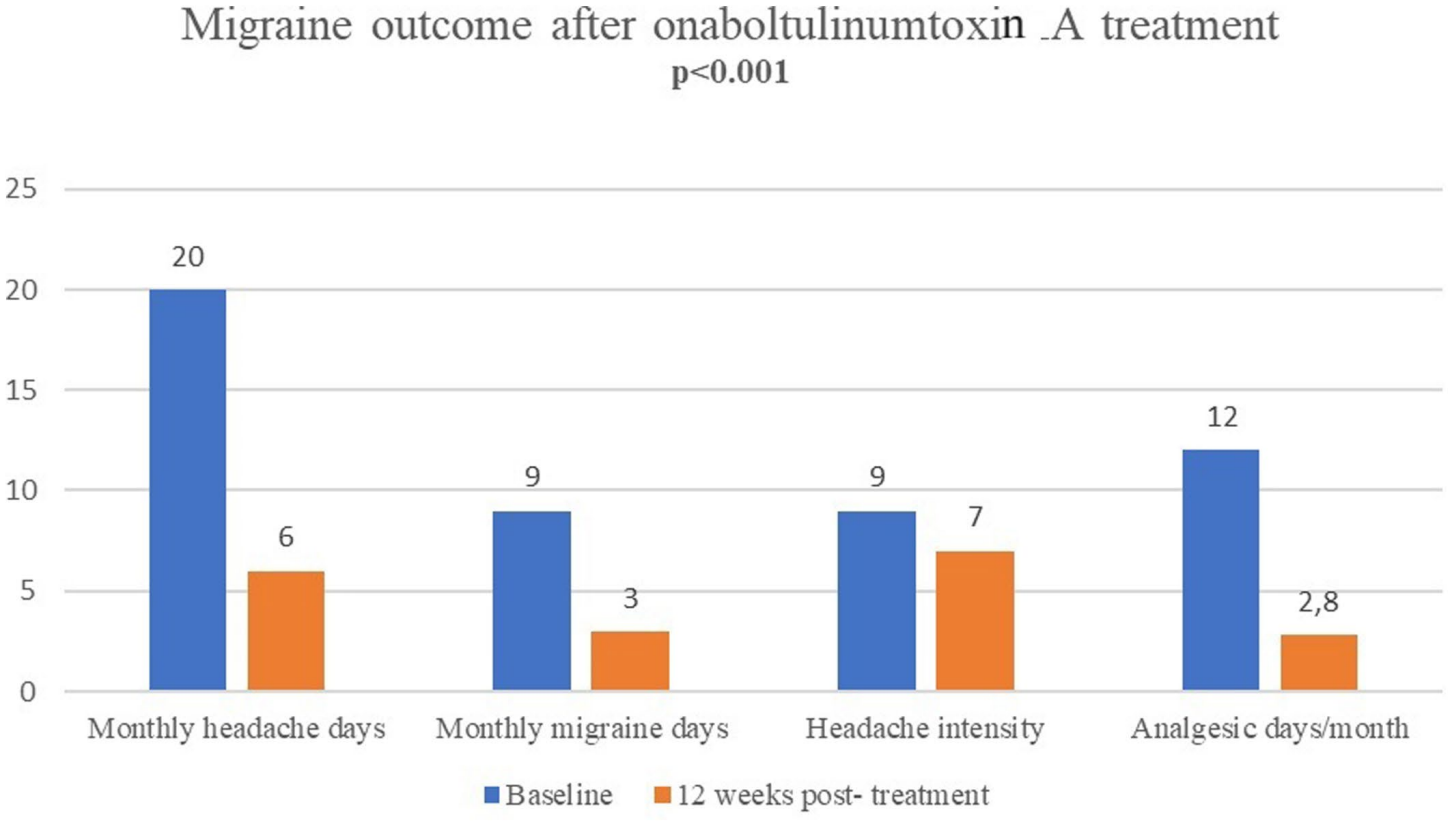
Migraine outcome in terms of monthly headache days, monthly migraine days, headache severity and monthly days of analgesic use after single-session onabotulinumtoxinA (at baseline vs. 12 weeks post-treatment).

OnabotulinumtoxinA revealed a significant decrease in median (IQR) MHDs (from 20(15–25) at baseline to 6(4–10), *p* < 0.001), MMDs (from 9(6–12) to 3(1–6), *p* < 0.001), headache severity NRS scores (from 9(8–10) to 7(6–8), *p* < 0.001) and migraine-specific (from 0(0–6.5) to 0(0–2), *p* = 0.010) and migraine non-specific (from 12(5.3–15) to 3.5(1–6.8), *p* < 0.001) analgesic use, at 12 weeks post-treatment (Table 2; Figure 2). Additionally, the number of patients overusing medications decreased from *n* = 35 (47.3%) to *n* = 11 (14.9%).

**Table 2:** Migraine outcome, accompanying symptoms.

	Baseline	12 weeks post-treatment	*p*-value
**Migraine outcome, median (IQR) (*n* = 72)**			
Monthly headache days	20(15–25)	6(4–10)	**<0.001** ^**1**^
Monthly migraine days	9(6–12)	3(1–6)	**<0.001** ^**1**^
Headache severity (NRS scores)	9(8–10)	7(6–8)	**<0.001** ^**1**^
**Duration of headache (hours)**			
*With analgesics*	3(1–10)	1(1–5)	0.137^1^
*Without analgesics*	24(7.3–60)	18(3.3–24)	0.075^1^
**Analgesic use per month**			
*Migraine non-specific (NSAIDs, paracetamol)*	12(5.3–15)	3.5(1–6.8)	**<0.001** ^**1**^
*Migraine specific (triptan, ergot, combined)*	0(0–6.5)	0(0–2)	**0.010** ^**1**^
**Accompanying symptoms, *n*(%)**			
Phonophobia (Ictal)	60 (83.3)	50 (69.4)	**0.013** ^**2**^
Osmophobia (Ictal)	45(62.5)	31 (43.1)	**0.003** ^**2**^
**Photophobia severity, median (IQR)**			
*Ictal*	5(4–5)	4(2–5)	**<0.001** ^**1**^
*Interictal*	2(1–3)	2(1–2)	**0.004** ^**1**^

NRS, Numeric rating scale; IQR, Interquartile range. ^1^Wilcoxon Signed Rank test; ^2^Mc Nemar test. The values in bold represent statistically significant differences.

Overall, 70.8% were considered responders (≥50% reduction of MHDs), and 29.2% of patients were non-responders (<50% reduction of MHDs). Also, 34.7% were super-responders (>75% reduction in MHDs).

### Accompanying features

A substantial decrease was noted in the rate of ictal phonophobia (from 83.3% to 69.4%, *p* = 0.013) and osmophobia (from 62.5% to 43.1%, *p* = 0.003) as well as in the severity of both ictal (from 5(4–5) to 4(2–5), *p* < 0.001) and interictal (from 2(1–3) to 2(1–2), *p* = 0.004) photophobia at 12 weeks post-treatment (Table 2).

### Patient-reported outcome measures

**Figure 3:**
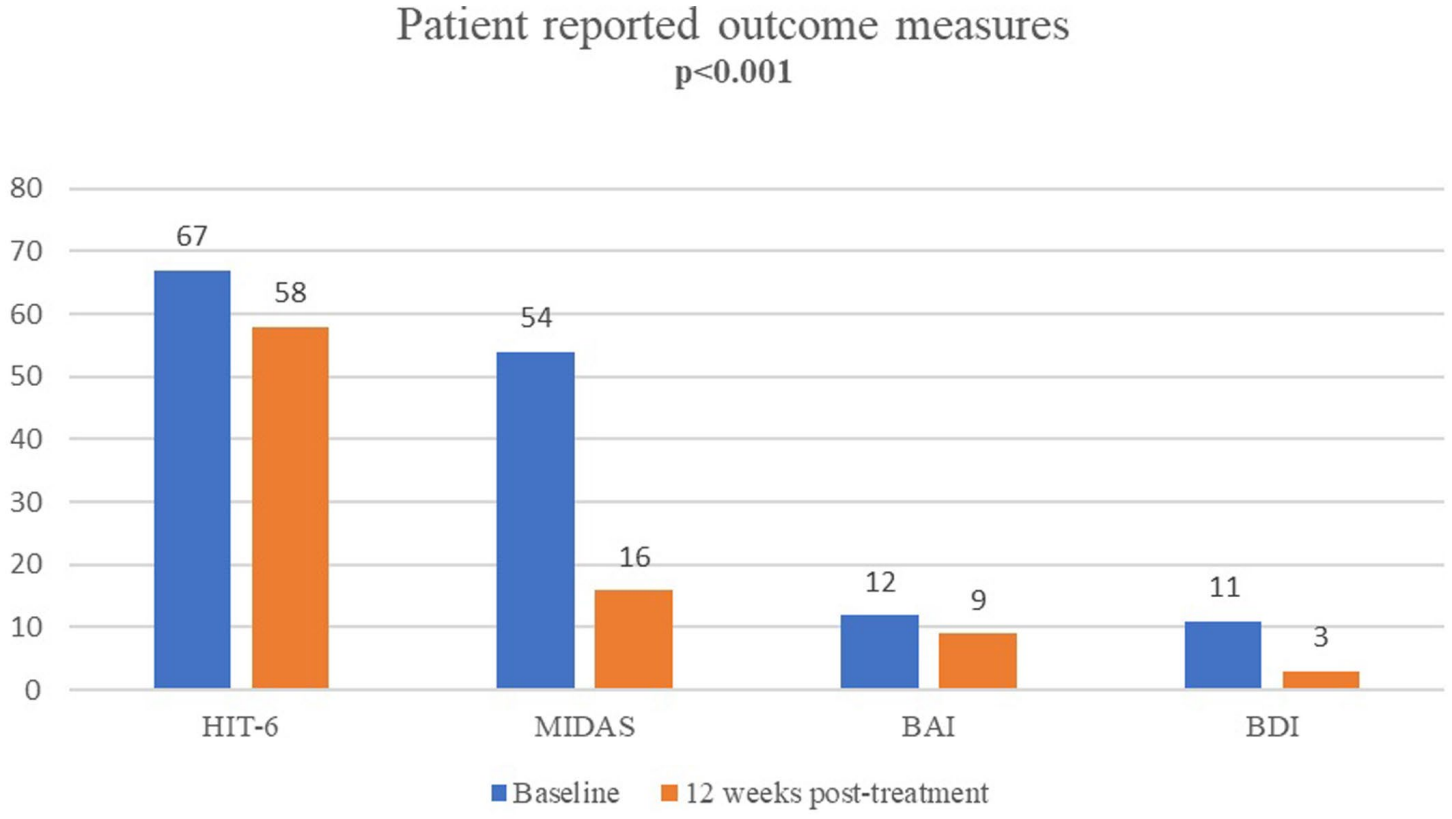
Patient-reported outcomes for migraine disability (MIDAS), impact (HIT-6), anxiety (BAI) and depression (BDI) after single-session onabotulinumtoxinA (at baseline vs. 12 weeks post-treatment).

The median(IQR) MIDAS scores (from 54(30–81) at baseline to 16(7–24) at 12 weeks, *p* < 0.001), HIT-6 scores (from 67(65–69) to 58(54–64), p < 0.001) and ASC-12 scores (from 6(1.5–9) to 2(0–9), *p* = 0.002) were significantly improved at 12 weeks post-treatment when compared to baseline scores (Table 3; Figure 3).

**Table 3:** Patient reported outcome measures.

Patients reported outcome measures (*n* = 72)	Baseline	12 weeks post-treatment	*p*-value
MIDAS score, median (IQR)	54(30–81)	16(7–24)	**<0.001**
HIT-6 score, median (IQR)	67(65–69)	58(54–64)	**<0.001**
ASC-12 score (ictal), median (IQR)	6(1.5–9)	2(0–9)	**0.002**
BAI score, median (IQR)	12(6.5–19)	9(3–17)	**<0.001**
BDI score, median (IQR)	11(6.5–17)	3(2–7)	**<0.001**

MIDAS, Migraine Disability Assessment Scale; HIT-6, Headache Impact Test-6; ASC-12, 12-item Allodynia Symptom Checklist; BAI, Beck Anxiety Inventory; BDI, Beck Depression inventory; IQR, Interquartile range. Mann Whitney U test. The values in bold represent statistically significant differences.

Median (IQR) BAI (from 12(6.5–19) to 9(3–17) *p* < 0.001) and BDI (from 11(6.5–17) to 3(2–7) *p* < 0.001) scores were significantly improved at 12 weeks post-treatment (Table 3; Figure 3).

### Masseter muscle injection and re-injection rate

At the initial visit, onabotulinumtoxinA was administered to 27(37.5%) patients with bruxism symptoms or findings, with a median dose of 30 IU. Of these patients, 19 (70.4%) who reported symptom improvement following the initial application received a re-injection during the second visit, with a mean dose of 40 IU.

### Baseline characteristics between responders and non-responders

The responder and non-responder groups were homogenous in terms of patient demographics, migraine history, headache characteristics, and accompanying symptoms, MOH, baseline scores for PROMs (MIDAS, HIT-6, ASC-12, BAI, and BDI), and the number of comorbidities and number of previous treatments (Table 4).

**Table 4:** Baseline characteristics in responder (≥50% reduction in MHDs) and non-responder (<50% reduction in MHDs) groups.

	Non-responders	Responders	*p*-value
**Demographic and migraine characteristics**			
Age (year), median(IQR)	34(30–39)	37(32–42)	0.095^1^
**Gender, *n*(%)**			
*Male*	1(4.8)	5(9.8)	0.664^2^
*Female*	20(95.2)	46(90.2)	
Duration of headache (years), median(IQR)	10(7.5–18)	14(4.7–25)	0.495^1^
Number of comorbidities, median(IQR)	2(1–3)	2(1–4)	0.624^1^
Monthly headache days, median(IQR)	16(15–20)	20(15–25)	0.135^1^
Headache intensity (NRS scores), median(IQR)	9(8–10)	8(8–10)	0.462^1^
**Duration of pain (hours), median (IQR)**			
*With analgesics*	3(1–16)	3(1–10)	0.968^1^
*Without analgesics*	24(7–48)	24(12.5–24)	0.370^1^
**Analgesic use days/per month, median (IQR)**			
*Migraine non-specific*	10(5.5–15)	12(5.5–17)	0.782^1^
*Migraine specific*	1(0–7)	0(0–5.5)	0.261^1^
Response to analgesics	3(2–4.5)	3(2.5–4)	0.828^1^
**Medication overuse headache, *n*(%)**			
*Present*	11(52.4)	24(47.1)	0.681^2^
*Absent*	10(47.6)	27(52.9)	
**Photophobia severity, median (IQR)**			
*Ictal*	5(3–5)	5(2–5)	0.305^1^
*Interictal*	3(1–5)	2(1–5)	0.481^1^
**Baseline PROMs scores, median(IQR)**			
MIDAS score	60(36–76)	50(30–84)	0.541^1^
HIT-6 score	68(65.5–70.5)	66(64–68)	0.298^1^
ASC-12 score (ictal)	6(2–10)	5(1–9)	0.538^1^
BAI score	11(5–19.5)	13(8–19)	0.375^1^
BDI score	8(5–14)	12(7–19)	0.283^1^
**Former treatments, median (IQR)**			
Overall number of former treatments	1(0–2)	1(0–1)	0.322^1^

NRS, Numeric rating scale; PROMs, patient-reported outcome measures; MIDAS, Migraine Disability Assessment Scale; HIT-6, Headache Impact Test-6; ASC-12, 12-item Allodynia Symptom Checklist; BAI, Beck Anxiety Inventory; BDI, Beck Depression Inventory. ^1^Mann Whitney U test, ^2^Fisher Exact test.

No notable distinctions were observed in the baseline BAI and BDI scores, and no alterations from baseline for MIDAS, HIT-6, and ASC-12 among individuals with a prior treatment history (Table 5).

**Table 5:** Comparison of patient-reported outcome measures between previously treated and untreated patients.

	Previous treatment		
	No	Yes	*p*-value
**Baseline scores, median (IQR)**			
BDI score	10(7–16)	11(5.2–20.5)	0.586^2^
BAI score	12(6–16.7)	13.5(7–19.7)	0.307^2^
MIDAS score	49(30–77)	52(30.7–78)	0.812^2^
HIT-6 score	67(65–68)	67(64–69)	0.860^2^
ASC-12 score (ictal)	5(2.2–10)	6(0.2–9)	0.200^2^
Monthly headache days	18(15–23.2)	20(15–25)	0.945^2^
Monthly migraine days	8.5(6–11.5)	9(6–15)	0.585^2^
Headache severity (NRS scores)	9(8–10)	9(8–10)	0.693^2^
**Change from baseline, median (IQR)**			
BAI score	−3(−10/−0.5)	−3.5(−6/1.2)	0.820^2^
BDI score	−6(−11/−2)	−4(−11.5/−1)	0.682^2^
MIDAS score	−27(−49.7/−17)	−32(−59/−12)	0.809^2^
HIT-6 score mean (± SD)	−7.67(± 9.03)	−9.55(± 7.18)	0.364^1^
ASC-12 score (ictal) (± SD)	−0.72(± 7.65)	−2.57 (± 3.83)	0.244^1^
Monthly headache days (± SD)	−11.34(± 7.37)	−12.4(± 7.48)	0.551^1^
Monthly migraine days	−4.5(−9/−2.2)	−6(−10/−2)	0.535^2^
Headache severity (NRS scores)	−1(−3/0)	−2(−4/0)	0.503^2^

BDI, Beck Depression Inventory; BAI, Beck Anxiety Inventory; MIDAS, Migraine Disability Assessment Scale; HIT-6, Headache Impact Test-6; ASC-12, 12-item Allodynia Symptom Checklist; NRS, Numeric Rating Scale. ^1^Student t test, ^2^Mann Whitney U test.

### Safety outcome

The TRAEs included headache in 9(12.5%) patients and pain at the injection site in 5(6.9%) patients, followed by neck pain, back pain, ptosis, and neck weakness (each in 1[1.4%] patient).

## Discussion

This real-world observational study in CM indicated the association of single-session onabotulinumtoxinA treatment with an improved migraine outcome (MHDs, MMDs, and analgesic use) and reduced migraine disability and impact. Furthermore, improvements were noted in headache intensity and the alleviation of associated symptoms, including allodynia, photophobia, osmophobia, and phonophobia, alongside a reduction in comorbid anxiety and depression.

Various pathways likely mediate the beneficial effects of onabotulinumtoxinA. It inhibits the release of pro-inflammatory and excitatory neurotransmitters such as substance P and CGRP from c-fibers endings, reducing peripheral sensitization. Additionally, it decreases the insertion of pain-sensitive ion channels into synaptic membranes, lowering sensory neuron excitability and inhibiting central sensitization involved in migraine pathophysiology (51–53).

The pivotal PREEMPT (Phase 3 Research Evaluating Migraine Prophylaxis Therapy) randomized controlled trials (RCTs) confirmed the efficacy of onabotulinumtoxinA in reducing MHDs and monthly migraine days (MMDs) with favorable safety and tolerability in CM patients (49, 50, 54, 55). A systematic review of real-world studies and RCTs related to the use of onabotulinumtoxinA in CM patients (26), found that the 28-day post-treatment change in MHDs (range, −7.4 to −14.7) (56–62) and MMDs (range, −9.4 to 11.9) (60, 61) aligned with the data from PREEMPT trials (−8.2 and −8.4, respectively) (49, 54, 55). Similarly, a meta-analysis of 44 real-world studies (25) indicated that outcomes at approximately 24 weeks and 52 weeks were broadly consistent with PREEMPT trials (54, 55) in terms of reducing MHDs, days of analgesics intake per month, and HIT-6 score (25).

Hence, our findings support the previous studies in the real-life setting confirming the benefits of onabotulinumtoxinA demonstrated in the RCTs and open-label studies of onabotulinumtoxinA, which involved several clinical parameters (i.e., monthly days of headache, acute medication intake, pain intensity, and migraine-related disability) (25, 26, 49, 50, 56–63). The changes in median MHDs (from 20 to 6), MMDs (from 9 to 3), headache intensity (from 9 to 7), MIDAS scores (from 54.0 to 16.0) and HIT-6 scores (from 67 to 58) at 12 weeks post-treatment in our cohort are in line with the previous onabotulinumtoxinA studies, while the onabotulinumtoxinA response rate was 70.8% (34.7% were super-responders) indicating the real-world effectiveness of onabotulinumtoxinA starting from the first session.

The reduction in median MIDAS scores and HIT-6 scores from baseline to 12 weeks post-treatment in our patients highlights the association of a single-session onabotulinumtoxinA treatment with significant enhancement of migraine disability (from severe to moderate disability status) and impact (from severe to substantial impact status). When combined with improvements in accompanying symptoms like photophobia, phonophobia, and osmophobia, onabotulinumtoxinA emerges as a comprehensive agent in migraine prophylaxis. Similarly, studies noted significant improvements in the HIT-6 and the MIDAS scores after 2 to 4 sessions of onabotulinumtoxinA (64, 65).

Nearly 30% of our patients were experiencing comorbid psychiatric disorders, with 19.4% having anxiety and 16.6% having depression. This aligns with the known association of CM with depression (up to 47%) and anxiety (up to 58%), and a fivefold increased risk of developing major depression compared to the general population (26, 66, 67). The prompt onset of improvement in our cohort’s BDI, BAI, and ASC-12 scores is crucial, given that depression, anxiety, and allodynia are suggested risk factors for migraine chronification, reduced treatment response, diminished quality of life, and heightened overall disease burden (8, 58, 68–70). Notably, findings from the real-world COMPEL study revealed that CM patients receiving 2 years of onabotulinumtoxinA experienced enhanced depressive symptoms even in the non-responders without a satisfactory reduction in headache days, emphasizing the likelihood of the direct effect of onabotulinumtoxinA on depression and anxiety (26, 71, 72).

Numerous studies investigate predictive markers for the effectiveness of onabotulinumtoxinA in chronic migraine patients. The presence of pericranial muscle tenderness was stated as a predictor for a higher probability for a good response to onabotulinumtoxinA in CM (33, 73). There are conflicting views on whether a good response to triptans could predict future outcomes. Some argue in favor of this notion (74), while others oppose it (75). While alleviation of CM after onabotulinumtoxinA decreases over 30 years of disease duration (35, 76), treatment within the first year after the diagnosis of CM may increase the chance for a better response (76).

In our cohort, both responders (70.8%) and non-responders (29.2%) exhibited similar baseline headache characteristics, comorbid diseases, days of analgesic use per month, migraine duration in years, and scores on patient-reported outcome measures (PROMs) like MIDAS, HIT-6, BDI, and BAI. Despite over half of the patients having received prior migraine prophylaxis there was no significant difference observed between previously treated and treatment-naive patients or between responders and non-responders to previous treatments and abortive medication in terms of MHDs and MMDs following onabotulinumtoxinA injection. In a study of 212 patients with CM and high-frequency episodic migraine receiving onabotulinumtoxinA no anamnestic characteristics differentiated responders from non-responders in the CM group (77). Similarly, a study by Pagola et al. failed to identify any clinical feature in patients with refractory migraine that predicts a favorable response to onabotulinumtoxinA treatment (78).

While onabotulinumtoxinA is usually initiated after the failure of at least three prior prophylactic agents in CM patients, by the current guideline recommendations (79–81), its efficacy is considered to be more significant when administered earlier in the course of CM (82, 83). Besides, a negative correlation was noted between the reduction in pain intensity and the number of previous drug treatments before the onset of onabotulinumtoxinA (82, 83). OnabotulinumtoxinA is widely regarded as an effective treatment for different types of migraine, including CM in patients with prior treatment failures, MOH, and comorbid depression and/or anxiety. It could be considered a first-line therapy for CM (26, 84). In our cohort, the one-third reduction in MOH rates at the second visit underscores the efficacy of onabotulinumtoxinA as a treatment option for this headache type, emphasizing the importance of early recognition of migraine chronification for timely initiation of effective prophylactic therapy and potentially better outcomes (85–87).

In our study, each patient was asked whether they experienced symptoms of teeth grinding and if they felt any discomfort associated with it. Additionally, a short examination included checking for linea alba at the buccal mucosa, masseter muscle hypertrophy and indentations along the tongue due to chronic repeated pressure. The first session of onabotulinumtoxinA injection was applied to the masseter muscles in 27 patients (37.5%). A majority (70.4%) of these gave positive feedback regarding decreased bruxism symptoms after 12 weeks, prompting a second injection of onabotulinumtoxinA. Despite significantly higher baseline BAI scores in patients with comorbid bruxism, the effectiveness of onabotulinumtoxinA on migraine outcomes was similar in patients with and without bruxism. In a double-blind, placebo-controlled study, participants experienced the largest reduction in the bruxism index at 4 weeks post-injection of onabotuliunmtoxinA compared with placebo, especially when injections were administered together to the temporal (each 15 IU) and masseter (each 30 IU) muscles. Furthermore, near our findings, 77% of participants asked for reinjection after 12 weeks (88). A meta-analysis indicated that injecting onabotuliunmtoxinA into the masseter, temporalis, and pterygoid muscles led to greater pain reduction than targeting only the masseter and temporalis muscles, after 6 months (89). A study demonstrated that onabotulinumtoxinA injections effectively lowered pain scores in the masseter muscles compared to conventional bruxism treatments (90), establishing it as a safe treatment option for bruxism with potential to prevent dental complications (91–93). OnabotulinumtoxinA demonstrated a positive impact on sleep quality in CM patients who did not exhibit negative emotional states (94). Employing masseter muscle onabotulinumtoxinA injections in patients with comorbid bruxism could offer additional value by alleviating bruxism as comorbidity alongside chronic migraine (93, 95, 96).

The safety profile and tolerability of onabotulinumtoxinA are considered excellent with rare, mild, and self-limiting TRAEs that rapidly and spontaneously resolve without complications (26, 97). Consistent with our findings, in a meta-analysis of RCTs on the safety of onabotulinumtoxinA, neck pain, musculoskeletal pain, muscular weakness, migraine, eyelid ptosis, blurred vision, and injection site pain were found to be the most common TRAEs, which were also mild-to-moderate in severity and resolved without sequelae (97).

Certain limitations to this study should be considered. First, despite providing data on real-life clinical practice, the potential lack of generalizability is an important limitation due to the single-center retrospective design of the study. Second, our findings provide data on single-session onabotulinumtoxinA therapy with likely changes in outcome measures and response rates in the consequent sessions.

Third, while polysomnography is the gold standard for diagnosing sleep bruxism, the diagnosis is often based on medical or dental history (98). Furthermore, the absence of data regarding the efficacy of masseter injections in treating bruxism within our cohort is an additional limitation. Also, enhancing our methodology by including the assessment of phonophobia and osmophobia during the interictal period could have provided further valuable information about the interictal burden in migraine in our cohort.

In conclusion, this real-world study revealed that onabotulinumtoxinA therapy was an effective treatment option with favorable safety profile in patients with CM, enabling rapid-onset improvements in migraine outcome (MHDs, MMDs, headache intensity, and analgesic use), migraine disability and impact, regardless of previous migraine prophylaxis history. Besides, the benefits of onabotulinumtoxinA were not limited to migraine outcomes but also involved a decrease in accompanying symptoms and the amelioration of comorbid depression and/or anxiety, allodynia, and possibly bruxism. Further real-world studies with longer follow-up in migraineurs, particularly in those with comorbidities, are needed to understand better the extent, durability, and predictors of response to onabotulinumtoxinA and to optimize its positioning within the current migraine prophylaxis practice.

## Data availability statement

The raw data supporting the conclusions of this article will be made available by the authors, without undue reservation.

## Ethics statement

The studies involving human participants were reviewed and approved by the Medical Research Ethics Committee of Acıbadem University (Approval number: 2023-21/726). Written informed consent from the patients/participants or patients/participants’ legal guardian/next of kin was not required to participate in this study in accordance with the national legislation and the institutional requirements.

## Author contributions

EI: Conceptualization, Data curation, Formal analysis, Investigation, Methodology, Project administration, Supervision, Writing – original draft, Writing – review & editing. TE: Data curation, Formal analysis, Investigation, Methodology, Writing – review & editing. PY: Data curation, Formal analysis, Investigation, Methodology, Supervision, Writing – review & editing.
